# Cuticular Compounds Bring New Insight in the Post-Glacial Recolonization of a Pyrenean Area: *Deutonura deficiens* Deharveng, 1979 Complex, a Case Study

**DOI:** 10.1371/journal.pone.0014405

**Published:** 2010-12-21

**Authors:** David Porco, Anne Bedos, Louis Deharveng

**Affiliations:** 1 Laboratoire Dynamique de la Biodiversité, UMR 5172, Université Paul Sabatier, Toulouse, France; 2 UMR 7205 CNRS “Origine, Structure et Evolution de la Biodiversité”, Muséum National d'Histoire Naturelle, Paris, France; Montreal Botanical Garden, Canada

## Abstract

**Background:**

In most Arthropod groups, the study of systematics and evolution rely mostly on neutral characters, in this context cuticular compounds, as non-neutral characters, represent an underexplored but potentially informative type of characters at the infraspecific level as they have been routinely proven to be involved in sexual attraction.

**Methods and Findings:**

The collembolan species complex *Deutonura deficiens* was chosen as a model in order to test the utility of these characters for delineating four infraspecific entities of this group. Specimens were collected for three subspecies (*D. d. deficiens*, *D. d. meridionalis*, *D. d. sylvatica*) and two morphotypes (*D. d. sylvatica* morphoype A and B) of the complex; an additional species *D. monticola* was added. Cuticular compounds were extracted and separated by gas chromatography for each individual. Our results demonstrate that cuticular compounds succeeded in separating the different elements of this complex. Those data allowed also the reconstruction of the phylogenetic relationships among them.

**Conclusions:**

The discriminating power of cuticular compounds is directly related to their involvement in sexual attraction and mate recognition. These findings allowed a discussion on the potential involvement of intrinsic and paleoclimatic factors in the origin and the diversification of this complex in the Pyrenean zone. This character type brings the first advance from pattern to process concerning the origin of this species complex.

## Introduction

Species and subspecies are taxonomic levels generally considered as objective descriptors of biological reality, a mere fact at the origin of the numerous different studies proposed to date. Whereas long diverged lineages are generally easy to separate with various sets of characters, recently diverged lineages are usually much more difficult to address. In those cases, classical morphological approaches sometimes fail to provide consistent discrimination or hypotheses about evolutionary history and relationships, and benefit from the inclusion of other markers with more appropriate and objectively measurable rates of divergence [1]. Besides the most commonly used neutral genetic markers such as ISSR [2], microsatellites [3] nuclear and mitochondrial sequences [4.5], allozymes [6] and karyotypes [7], [8], alternative sets of characters, such as cuticular compounds, have been proposed and tested with promising results [9], [10]. These compounds are lipids present on the most external cuticular layer of all terrestrial arthropods: the epicuticular layer. This layer is composed of various categories of lipids. In most cases, the majority of these compounds are hydrocarbons but other types of compounds can be present such as fatty acids, ketones, alcohols and esters [11], [12]. As the benefit of using the maximum number of compounds for population level studies has already been emphasized in the literature [10], in the present study we consider the total composition of the cuticular profile regardless of families to which the chemical compounds belong. In insects, the different types of cuticular compounds are functionally involved, particularly in sexual communication [13], [14]. In Collembola, intraspecific interactions such as reproductive attraction and recognition signalling [15], [16], aggregation [17], [18], [19], [20], [21] and alarm [22], [23], [24] rely heavily on olfactory interactions through chemical compounds.

As they also play a major role in sexual recognition [25], [26] and thus in premating isolation mechanisms [27], cuticular compounds, as a type of character, deserve a special attention in the study of groups of subspecific entities. This is especially relevant for Collembola since cuticular compounds were proven to play a major role in sexual recognition [16] suggesting that they could be potential key drivers of reproductive isolation, making them good candidates for infraspecific taxon discrimination in this group.

A previous population study of the wingless grasshopper *Chorthippus parallelus* from the Pyrenees revealed significant differences between subspecies cuticular profiles and suggested their major implication in specific sexual recognition [28]. A quantitative sexual dimorphism in cuticular blends was found in this grasshopper species [10], [29] and subsequently those compounds were proven to be involved in reproductive isolation mechanisms [30]. A similar sexual dimorphism was also demonstrated in Collembola [31].

In the present study, we tested the discriminating power of such characters at the infraspecific level for Collembola using the *Deutonura deficiens* complex as a model. This thoroughly studied group displays a very complex pattern of closely related subspecies and morphotypes living in parapatry within a relatively limited geographic range. Narrow overlapping areas and hybrid zones were documented in the Pyrenees region [32], [33]. This complex includes three subspecies: *D. d. deficiens*, *D. d. meridionalis* and *D. d. sylvatica*. Each one splits into several morphotypes that are geographically well delimited. These infraspecific taxonomic categories defined by Deharveng [32] are based on their distribution and morphological similarity within populations (cephalic tuberculisation among subspecies and abdominal setae and body pigmentation for morphotypes within subspecies).

The extensive knowledge of the repartition areas of these different infraspecific entities allowed an overall assessment of the role that ecological factors played in producing the current biogeographical pattern and the position of the contact zones [32]. None of the ecological factors examined (vegetation, altitude, precipitation, temperature or hydrographic network) were found to be significantly related to the species boundaries within the mosaic pattern [33]. Concerning the origin of the pattern, these results suggest the involvement of inherent characteristics of the organisms and/or an historical inheritance.

In this context we address the utility of cuticular compounds as a set of characters for (1) separating infraspecific lineages in Collembola and (2) establishing phylogenetic relationships between them. We also discuss the origin of the parapatric pattern in the context of European glacial history.

## Materials and Methods

### Samples

Specimens were collected in the field, either by direct capture on substrate or by extraction from litter and decayed wood with Berlese funnels. They were freeze killed and stored at -28°C. In order to avoid the potential bias of variation of the cuticular profile with age [34], only adult individuals were used. In this study, 21 individuals from the *D. d. deficiens* subspecies, 13 from *D. d. meridionalis* and 21 from *D. d. sylvatica* (with 12 individuals from morphotype C and 9 from morphotype A) were analysed (Table 1). In order to infer phylogenetic relationships among these infraspecific entities, we used an external species, *Deutonura monticola* (17 individuals), to root the evolutionary hypothesis. Identifications to subspecies and morphotypes were carried out through morphological examination of specimens after microscopic preparation of all individuals.

**Table 1:** Size and locations of sampled populations.

Species	N	Collection sites/date
*Deutonura deficiens deficiens*	21	Bouconne forest (Haute Garonne) 2003
*Deutonura deficiens sylvatica* Morphotype A	9	Caves of Lacave (Lot) 2002
*Deutonura deficiens sylvatica* Morphotype C	12	Caves of Lacave (Lot) 2002
*Deutonura deficiens meridionalis*	13	Montesquieux des Albères (Pyrénées Orientales) 2002
*Deutonura monticola*	17	Renne les Bains (Aude) 2003

### Gas Chromatography

Cuticular lipids were extracted by soaking each individual for 5 minutes in 20 µl of hexane. Separation of compounds in the extracts was performed on a Hewlett Packard Chromatograph model 5890 equipped with a Flame Ionization Detector (320°C) and an automatic sampler (HP 6890). For each individual, 5 µl of extract was injected in an apolar capillary column (HT-5) of 25m long. Helium was used as the carrier gas (pressure 16 psi - injector temperature 280°C). The temperature program started from 150°C and reached 320°C with a rate of 5°C/min. Then a 20 minutes 320°C isotherm was used to clean up the column between two samples. Each compound was characterized with its Kováts index (KI) [35] calculated using the coelution of samples with a standard solution of linear alkanes (C16 to C40) (Table S1). This homology hypothesis relying on KI brings limitations because compounds were not structurally identified. Some compounds even if structurally different can have the same KI. In some cases, this could lead to an underestimation of the variability of the profiles.

### Statistical Analysis

The data were recovered with the Millennium 2.15 software (Waters). For each individual, the relative surfaces of each peak in the chromatogram matched the concentration of the corresponding cuticular compound in the blend. Those relative surfaces were calculated and the data were exported to Systat 10.0 for statistical treatment. A stepwise forward discriminant analysis was performed to explore infraspecific variation, and the “F-to-remove” value was retained for each discriminant peak. This value is a good indicator of peak importance for distinction between groups. But a discriminant analysis alone can eliminate highly correlated values and thus give a partial view of the data set separating different classes. For this reason we also performed Pearson Correlation Matrix with a Bonferroni correction (P<0.005) to recover the correlated peaks (Table S1 - Supplementary material). A jackknife resampling method (default for discriminant analysis in the SYSTAT 10.0 software) was used to evaluate the discriminatory power of the cuticular compounds.

### Phylogenetic Reconstruction

Distance relations were established between the different infraspecific entities through a Neighbor Joining calculation with MEGA 4 [36]. The Mahalanobis distances matrix obtained from the previous discriminant analysis (i.e. distances between centroïds of the different infra-specific entities) was used for this purpose (Table 2). The external species *D. monticola* was used to root the phylogenetic hypothesis.

**Table 2:** Mahalanobis distances matrix obtained via the discriminant analysis.

	*D. d. sylvatica* morphotype C	*D. d. sylvatica* morphotype A	*D. d. deficiens*	*D. d. meridionalis*	*D. monticola*
*D. d. sylvatica* morphotype C	0				
*D. d. sylvatica* morphotype A	24.572	0			
*D. d. deficiens*	38.978	91.873	0		
*D. d. meridionalis*	34.02	66.628	11.49	0	
*D. monticola*	49.348	33.383	131.278	71.982	0

## Results

**Figure 1:**
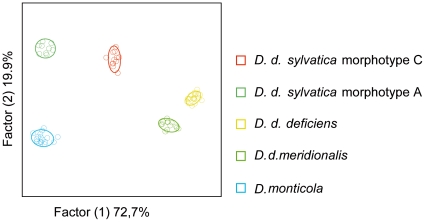
Graphic representation of the discriminant analysis following the two first axis accounting for 92.6% of the variability.

A total of 128 distinct compounds were found for *D. deficiens* subspecies, morphotypes, and the external species *Deutonura monticola*. After the statistical analysis, we obtained a significant discrimination (P<0.00001, F = 27.6126) between the four infra-specific taxa and *D. monticola* (Fig. 1). The Jacknife classification value was 100% for all taxa but *D. d. sylvatica* morphotype C with one individual misplaced (Table 3). Thirty-five cuticular compounds were designated as discriminant. Eighty-two other compounds were recovered using the correlation matrix. Eventually, 117 products allowed separation of the groups (Table S1 - Supplementary material). All variations were quantitative. Most compounds are implicated in the discrimination, indicating profile proximity between the different morphotypes and subspecies with numerous quantitative variations at a fine scale.

**Table 3:** Jackniffe assignation values of the discriminant analysis for each infraspecific entity.

	*D. d. sylvatica* morphotype C	*D. d. sylvatica* morphotype A	*D. d. deficiens*	*D. d. meridionalis*	*D.monticola*	% correct assignation
*D. d. sylvatica* morphotype C	12	0	0	0	0	100
*D. d. sylvatica* morphotype A	0	9	0	0	0	100
*D. d. deficiens*	1	0	20	0	0	95
*D. d. meridionalis*	0	0	0	13	0	100
*D.monticola*	0	0	0	0	17	100
Total	13	9	20	13	17	99

**Figure 2:**
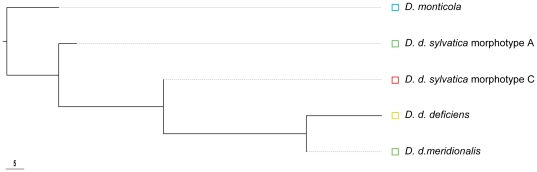
Phylogenetic relationships between subspecies and morphotypes of *Deutonura deficiens* build from the Mahalanobis distances rooted with *D. monticola*.

Phylogenetic relationships were established and rooted with *Deutonura monticola* (Fig. 2). The subspecies *D. d. sylvatica* appeared as the first derived form of the lineage with its two morphotypes aggregated in basal position. Then the two other subspecies, *D. d. deficiens* and *D. d. meridionalis*, branched together suggesting their later individualisation.

## Discussion

During the last twenty years, cuticular compounds have been investigated in chemotaxonomy for species specificity [37], [38], [39], [40], [41]. But the systematic use of those characters for taxonomy was challenged by some findings in termites: if cuticular compounds were proven useful in this group for species delimitation [40], [42], a strong variation of the profiles was found among several colonies of the same species [43], [44], [45]. But this is the peculiar case of social insect in which conspecific colonies are competing with each other and thus have a strong pressure for chemical recognition cues among colonies [13], [46], [47] even if a ‘dear enemy’ effect was demonstrated in termites [48]. It was suggested that a part of the cuticular compounds can be incorporated from some of the substances found in the alimentation as a possible explanation for the high variability of profiles among conspecific colonies of termites [48], such a phenomenon was already proven to occur in ants [49].

By contrast there is not such a competitive pressure among individuals in solitary animals like collembolans and thus a selection pressure toward a strong individualization of their cuticular profiles cannot be expected. Furthermore the repartition areas of the infraspecific entities of the species complex studied in this paper are very restricted and so the variations of feeding possibilities are expected to be quite narrow on such a range where the types of habitats are similar. The low variability of the collembolans cuticular profile within and among different populations of the species was documented in this respect [50] which allows to push the test of the taxonomic resolution further here.

In this study, cuticular compounds successfully discriminated all of the infraspecific taxa assigned by morphology within the *Deutonura deficiens* complex. The congruence between the two types of characters supports the biological reality of those entities. In addition, they brought further understanding of the phylogenetic relationships among these taxa. Those data confirmed that in Collembola, this character type is phylogenetically informative at low taxonomical levels [50].

The phylogenetic relationships established from cuticular profiles support two hypotheses: (a) an early individualization of *D. d. sylvatica* morphotypes A and C, which is consistent with the current sympatry of some of their populations exhibiting a high density of both parental phenotypes and an absence of hybrids; such a pattern and their basal position in the chemical phylogeny support the hypothesis of an achieved speciation phase with pre-reproductive isolation completed as hypothesized by Deharveng [32], (b) a more recent appearance of *D. d. deficiens* and *D. d. meridionalis*; the strict parapatry and the occurrence of morphologically intermediate specimens (assumed to be hybrids) among mixed populations in narrow contact zones between those two subspecies provides further support for their recent individualization.

As these chemicals play a crucial role in sexual attraction and mate recognition in Collembola [16], the discrepancies found among the cuticular profiles of all the infraspecific entities of the *D. deficiens* complex strongly suggest a status of an ongoing process of reproductive isolation involving those compounds. Clear divergence of cuticular profiles between sexes was demonstrated for other Collembola species [31]. Such sexual dimorphism exists also in *D. deficiens*, and all but one of the cuticular compounds involved in the sexual variation were implicated in the subspecies discrimination (Table S2 - Supplementary material). This indicates that sexual selection is mostly responsible for the variation of these traits, implying a major role of the cuticular compounds in sexual and specific recognition, which fits with the last findings in this field [16]. Then the possibility of a reinforcement phenomenon weighting on these characters as described by Higgie and Blows [51] could be considered.

The elements brought by the analysis of cuticular profiles suggest that several events of allopatry could have led to different levels of reproductive isolation responsible for the appearance of the distinct intraspecific entities and the origin of the current parapatric pattern of the *D. deficiens* complex. Pleistocene glacial events could be at the origin of the occurrence of allopatric phases for several populations of this species. A comparable mosaic distribution of the subspecies of another Collembola species of the same subfamily (*Monobella grassei*) was uncovered in the same region [52]. One of the hypothetical causes examined for such a pattern was the major historical event of the Pleistocene glaciations. These key paleoclimatic episodes are mainly accountable for the current repartition of animal and plant species populations: a correlation was established between variation in vegetal cover and climatic osrelationships were established cillations in the last 135 000 years [53], and Hewitt [54] proposed three European models for post-glacial recolonization in the Pleistocene based on plant and animal cases. The knowledge of the geographic distribution of the taxa studied here allows a comparison with Hewitt's recolonization models [54] in order to discuss the origin of the actual pattern (described in [33]) in a historical perspective. One of the postglacial recolonization models described by Hewitt [54] matches the existing distribution of the *D. deficiens* subspecies and the location of the contact zones (the recent discovery of a *D. d. sylvatica* population in the Italian Peninsula strengthens this support). It implies a faster advance of the populations from eastern refuges (Balkan, southern Carpathians and Caucasus) by contrast with the populations from the Iberic and Italian Peninsula refuges. As a result, the northern zones of these southern refuges were colonized by eastern populations blocking the advance of the southern ones and leading to the occurrence of contact zones. The current repartition and the contact zones position observed in the *D. deficiens* complex is congruent with the scenario of post-glacial recolonization exhibited by several taxa [54]. This brings support to the involvement of the last Pleistocene glacial events in the establishment of this distribution pattern.

Though we acknowledge some limitations in the understanding of some aspects of the complex wider geographic repartition and history: the possible occurrence of populations belonging to *D. deficiens* complex in eastern refuges, or the role played by Atlantic and Mediterranean microhabitats near actual contact zones which could have interfered with this model at fine scales as described in the ‘refugia within refugia’ concept developed for the Balkan Peninsula [55].

The successful use of a type of character directly involved in the mate choice on genetically distinct but closely related entities in a parapatric context, underlies their predominance in the origin and the maintenance of this parapatric pattern. The complexity of the whole pattern calls for a comparison of other markes types and a more exhaustive sampling through existing and potential repartition areas of the complex. The elucidation of the origin and the maintenance of such a pattern is crucial to understand what has driven the repartition and diversity of understudied soil microarthropods in this critical European zone.

## Supporting Information

Table S1 Discriminant analysis results for the 4 infraspecific entities and the external species *D. monticola*.
(0.24 MB DOC)

Table S2 Discriminant analysis results between sexes in *D. d. meridionalis* (P<0.00001, F = 193,3723; Jackknife 100%).
(0.03 MB DOC)
