# Supplementary material for: Cuticular Compounds Bring New Insight in the Post-Glacial Recolonization of a Pyrenean Area: Deutonura deficiens Deharveng, 1979 Complex, a Case Study
Source: PLoS One. 2010 Dec 21;5(12):e14405. doi: 10.1371/journal.pone.0014405 (PMC3014355; doi:10.1371/journal.pone.0014405)
Supplement: Table S1 — Discriminant analysis results for the 4 infraspecific entities and the external species D. monticola. (0.24 MB DOC) [file pone.0014405.s001.doc]

Table S1. Discriminant analysis results for the 4 infraspecific entities and the external species *D. monticola*

|  | |  | |  | |  |  | |  | | |  | |  | |  | |  |  |  |  |
| --- | --- | --- | --- | --- | --- | --- | --- | --- | --- | --- | --- | --- | --- | --- | --- | --- | --- | --- | --- | --- | --- |
|  |  | |  | |  |  |  |  | |  |  | |  | |  | |  | | | |  |
| **Compounds #** | **Kováts' index** | | **"F-to-remove"** | | **Mean relative surface in *D. d. deficiens*** | **Standard Error** | **Mean relative surface in *D. d. meridionalis*** | **Standard Error** | | **Mean relative surface in *D. d. sylvatica* morphotype A** | **Standard Error** | | **Mean relative surface in *D. d. sylvatica* morphotype C** | | **Standard Error** | | **Mean relative surface in *D. monticola*** | | | | **Standard Error** |
| 1 | 1638 | |  | | 0 | 0 | 0 | 0 | | 0 | 0 | | 0 | | 0 | | 0.127 | | | | 0.023 |
| 2 | 1723 | | 19.69 | | 0.121 | 0.023 | 0.037 | 0.01 | | 0.049 | 0.015 | | 0.201 | | 0.037 | | 0.05 | | | | 0.008 |
| 3 | 1745 | |  | | 0.135 | 0.015 | 0.131 | 0.025 | | 0.114 | 0.026 | | 0.127 | | 0.012 | | 0.073 | | | | 0.01 |
| 4 | 1756 | | 7.17 | | 0.664 | 0.062 | 0.507 | 0.086 | | 1.01 | 0.155 | | 0.702 | | 0.059 | | 0.442 | | | | 0.026 |
| 5 | 1821 | | 26.07 | | 1.852 | 0.239 | 3.56 | 0.369 | | 5.021 | 0.444 | | 5.793 | | 0.457 | | 4.284 | | | | 0.284 |
| 6 | 1834 | | 17.43 | | 0.465 | 0.062 | 0.437 | 0.058 | | 0.502 | 0.054 | | 0.65 | | 0.063 | | 0.434 | | | | 0.046 |
| 8 | 1858 | |  | | 1.21 | 0.189 | 1.038 | 0.125 | | 1.642 | 0.223 | | 1.977 | | 0.19 | | 1.405 | | | | 0.113 |
| 10 | 1938 | | 5.09 | | 0.65 | 0.144 | 0.308 | 0.094 | | 0.709 | 0.194 | | 0.789 | | 0.118 | | 0 | | | | 0 |
| 12 | 1950 | |  | | 1.013 | 0.172 | 0.781 | 0.104 | | 1.055 | 0.097 | | 1.229 | | 0.413 | | 0 | | | | 0 |
| 14 | 2025 | |  | | 0.155 | 0.03 | 0.213 | 0.033 | | 0.218 | 0.044 | | 0.169 | | 0.047 | | 0.409 | | | | 0.042 |
| 15 | 2034 | | 6.69 | | 0.471 | 0.067 | 0.234 | 0.027 | | 0.45 | 0.065 | | 0.438 | | 0.046 | | 0.879 | | | | 0.233 |
| 16 | 2043 | | 7.7 | | 1.883 | 0.188 | 1.835 | 0.316 | | 3.031 | 0.366 | | 2.471 | | 0.21 | | 1.04 | | | | 0.166 |
| 17 | 2058 | | 6.72 | | 0 | 0 | 0 | 0 | | 0 | 0 | | 0 | | 0 | | 0.327 | | | | 0.046 |
| 18 | 2069 | | 17.45 | | 0.372 | 0.085 | 0.151 | 0.022 | | 0.471 | 0.067 | | 0.675 | | 0.166 | | 0.33 | | | | 0.036 |
| 19 | 2102 | |  | | 0 | 0 | 0 | 0 | | 0 | 0 | | 0 | | 0 | | 0.101 | | | | 0.013 |
| 20 | 2125 | | 18.5 | | 0.832 | 0.212 | 2.936 | 0.903 | | 2.263 | 1.078 | | 3.446 | | 2.001 | | 3.922 | | | | 0.638 |
| 21 | 2143 | | 9.04 | | 13.264 | 2.104 | 20.728 | 2.134 | | 13.669 | 1.609 | | 19.04 | | 1.663 | | 23.271 | | | | 1.668 |
| 22 | 2148 | |  | | 10.475 | 0.925 | 13.372 | 0.937 | | 17.036 | 1.536 | | 14.425 | | 1.601 | | 8.915 | | | | 0.537 |
| 23 | 2152 | | 20.84 | | 5.661 | 0.507 | 7.095 | 0.629 | | 12.508 | 2.043 | | 9.493 | | 1.206 | | 6.397 | | | | 0.347 |
| 24 | 2172 | | 15.67 | | 6.882 | 0.772 | 6.518 | 0.506 | | 6.478 | 0.322 | | 7.891 | | 0.833 | | 6.172 | | | | 0.374 |
| 25 | 2214 | |  | | 0 | 0 | 0 | 0 | | 0 | 0 | | 0 | | 0 | | 0.32 | | | | 0.086 |
| 26 | 2223 | |  | | 0 | 0 | 0 | 0 | | 0 | 0 | | 0 | | 0 | | 0.465 | | | | 0.056 |
| 27 | 2226 | |  | | 0 | 0 | 0 | 0 | | 0 | 0 | | 0 | | 0 | | 0.776 | | | | 0.129 |
| 28 | 2243 | | 4.39 | | 0.355 | 0.089 | 0.309 | 0.053 | | 1.366 | 0.423 | | 0.623 | | 0.175 | | 0.204 | | | | 0.031 |
| 29 | 2249 | | 3 | | 1.726 | 0.315 | 1.496 | 0.233 | | 0.772 | 0.163 | | 0.26 | | 0.045 | | 0.04 | | | | 0.01 |
| 30 | 2270 | | 8.24 | | 0.434 | 0.068 | 0.31 | 0.101 | | 0.177 | 0.03 | | 0.267 | | 0.091 | | 0 | | | | 0 |
| 31 | 2276 | |  | | 0.091 | 0.018 | 0.07 | 0.023 | | 0.078 | 0.03 | | 0.436 | | 0.335 | | 0 | | | | 0 |
| 32 | 2301 | | 7.93 | | 3.378 | 0.521 | 2.827 | 0.306 | | 4.195 | 0.745 | | 2.991 | | 0.322 | | 6.121 | | | | 0.392 |
| 34 | 2336 | | 6.26 | | 0 | 0 | 0 | 0 | | 0 | 0 | | 0 | | 0 | | 0.858 | | | | 0.137 |
| 35 | 2342 | |  | | 0.624 | 0.099 | 0.829 | 0.171 | | 1.078 | 0.089 | | 1.47 | | 0.229 | | 0.752 | | | | 0.085 |
| 36 | 2349 | | 17.82 | | 0.638 | 0.056 | 0.67 | 0.088 | | 0.554 | 0.058 | | 0.461 | | 0.058 | | 0 | | | | 0 |
| 37 | 2357 | | 33.69 | | 0.176 | 0.028 | 0.131 | 0.046 | | 0.454 | 0.043 | | 0.351 | | 0.046 | | 0 | | | | 0 |
| 38 | 2377 | |  | | 0.206 | 0.03 | 0.246 | 0.029 | | 0.204 | 0.028 | | 0.168 | | 0.027 | | 0.097 | | | | 0.018 |
| 39 | 2384 | |  | | 0.193 | 0.028 | 0.218 | 0.027 | | 0.22 | 0.039 | | 0.128 | | 0.022 | | 0.1 | | | | 0.023 |
| 40 | 2393 | |  | | 0 | 0 | 0 | 0 | | 0 | 0 | | 0 | | 0 | | 0.058 | | | | 0.016 |
| 41 | 2414 | |  | | 0.094 | 0.018 | 0.103 | 0.034 | | 0.024 | 0.003 | | 0.047 | | 0.009 | | 0.068 | | | | 0.006 |
| 42 | 2425 | | 2.71 | | 0 | 0 | 0 | 0 | | 0 | 0 | | 0 | | 0 | | 0.035 | | | | 0.004 |
| 43 | 2436 | |  | | 0 | 0 | 0 | 0 | | 0 | 0 | | 0 | | 0 | | 0.028 | | | | 0.01 |
| 44 | 2447 | |  | | 0.283 | 0.054 | 0.218 | 0.05 | | 0.157 | 0.03 | | 0.247 | | 0.11 | | 0.291 | | | | 0.131 |
| 45 | 2452 | |  | | 4.451 | 1.056 | 2.847 | 0.569 | | 1.407 | 0.268 | | 1.941 | | 1.085 | | 1.742 | | | | 0.344 |
| 46 | 2455 | | 6.45 | | 1.214 | 0.196 | 0.924 | 0.146 | | 0.762 | 0.155 | | 0.99 | | 0.531 | | 1.023 | | | | 0.209 |
| 47 | 2473 | |  | | 0.15 | 0.029 | 0.226 | 0.037 | | 0.15 | 0.016 | | 0.203 | | 0.055 | | 0.054 | | | | 0.01 |
| 48 | 2481 | | 2.9 | | 0.235 | 0.035 | 0.17 | 0.024 | | 0.105 | 0.021 | | 0.102 | | 0.019 | | 0.169 | | | | 0.037 |
| 49 | 2486 | |  | | 0.095 | 0.014 | 0.137 | 0.014 | | 0.067 | 0.011 | | 0.043 | | 0.006 | | 0.048 | | | | 0.006 |
| 50 | 2505 | |  | | 0.1 | 0.018 | 0.128 | 0.022 | | 0.094 | 0.02 | | 0.072 | | 0.015 | | 0 | | | | 0 |
| 52 | 2523 | |  | | 0.421 | 0.134 | 0.17 | 0.057 | | 0.085 | 0.02 | | 0.061 | | 0.009 | | 0.025 | | | | 0.008 |
| 53 | 2531 | |  | | 0.086 | 0.036 | 0.031 | 0.004 | | 0.019 | 0.005 | | 0.021 | | 0.005 | | 0 | | | | 0 |
| 54 | 2540 | |  | | 0 | 0 | 0 | 0 | | 0 | 0 | | 0 | | 0 | | 0.035 | | | | 0.013 |
| 55 | 2554 | |  | | 0.071 | 0.022 | 0.053 | 0.01 | | 0.044 | 0.013 | | 0.035 | | 0.008 | | 0.03 | | | | 0.011 |
| 56 | 2595 | |  | | 0.274 | 0.048 | 0.127 | 0.016 | | 0.244 | 0.054 | | 0.13 | | 0.061 | | 0.138 | | | | 0.024 |
| 57 | 2603 | | 24.76 | | 0.782 | 0.177 | 0.256 | 0.046 | | 0.848 | 0.209 | | 0.375 | | 0.11 | | 0.202 | | | | 0.042 |
| 58 | 2606 | | 6.73 | | 0 | 0 | 0 | 0 | | 0 | 0 | | 0 | | 0 | | 0.039 | | | | 0.005 |
| 59 | 2613 | |  | | 0.064 | 0.01 | 0.05 | 0.006 | | 0.023 | 0.005 | | 0.027 | | 0.007 | | 0.039 | | | | 0.013 |
| 61 | 2642 | | 3.23 | | 0.039 | 0.006 | 0.047 | 0.011 | | 0.016 | 0.004 | | 0.022 | | 0.008 | | 0 | | | | 0 |
| 62 | 2658 | |  | | 0.202 | 0.029 | 0.148 | 0.014 | | 0.09 | 0.02 | | 0.071 | | 0.02 | | 0.119 | | | | 0.026 |
| 63 | 2661 | |  | | 0.068 | 0.008 | 0.07 | 0.006 | | 0.042 | 0.011 | | 0.034 | | 0.005 | | 0.06 | | | | 0.012 |
| 64 | 2680 | | 4.27 | | 0.077 | 0.011 | 0.045 | 0.005 | | 0.049 | 0.013 | | 0.026 | | 0.008 | | 0.079 | | | | 0.017 |
| 65 | 2687 | |  | | 0.312 | 0.204 | 0.049 | 0.017 | | 0.167 | 0.043 | | 0.053 | | 0.028 | | 0.06 | | | | 0.011 |
| 66 | 2699 | |  | | 0.123 | 0.027 | 0.059 | 0.01 | | 0.02 | 0.009 | | 0.05 | | 0.027 | | 0.039 | | | | 0.008 |
| 67 | 2695 | |  | | 0 | 0 | 0 | 0 | | 0 | 0 | | 0 | | 0 | | 0.072 | | | | 0.016 |
| 68 | 2713 | |  | | 0.11 | 0.023 | 0.095 | 0.045 | | 0.075 | 0.03 | | 0.046 | | 0.01 | | 0.074 | | | | 0.012 |
| 69 | 2755 | |  | | 0.036 | 0.009 | 0.095 | 0.069 | | 0.019 | 0.008 | | 0.031 | | 0.017 | | 0.118 | | | | 0.016 |
| 70 | 2800 | |  | | 0.482 | 0.059 | 0.269 | 0.089 | | 1.076 | 0.52 | | 0.159 | | 0.041 | | 0.605 | | | | 0.176 |
| 71 | 2817 | |  | | 0.038 | 0.007 | 0.1 | 0.075 | | 0.014 | 0.005 | | 0.038 | | 0.024 | | 0.04 | | | | 0.01 |
| 72 | 2830 | |  | | 0.166 | 0.03 | 0.306 | 0.223 | | 0.069 | 0.019 | | 0.037 | | 0.012 | | 0.094 | | | | 0.023 |
| 73 | 2847 | |  | | 0.04 | 0.007 | 0.131 | 0.105 | | 0.019 | 0.007 | | 0.303 | | 0.292 | | 0.029 | | | | 0.005 |
| 74 | 2880 | |  | | 9.477 | 1.325 | 5.624 | 0.692 | | 2.626 | 0.641 | | 2.522 | | 0.954 | | 5.648 | | | | 0.914 |
| 75 | 2903 | |  | | 1.825 | 0.743 | 0.726 | 0.192 | | 0.163 | 0.037 | | 0.153 | | 0.054 | | 0.595 | | | | 0.105 |
| 76 | 2918 | |  | | 0.094 | 0.044 | 0.202 | 0.185 | | 0.017 | 0.004 | | 0.011 | | 0.003 | | 0 | | | | 0 |
| 77 | 2928 | |  | | 0.219 | 0.062 | 0.17 | 0.14 | | 0.026 | 0.006 | | 0.037 | | 0.024 | | 0.036 | | | | 0.01 |
| 78 | 2937 | |  | | 0 | 0 | 0 | 0 | | 0 | 0 | | 0 | | 0 | | 0.16 | | | | 0.028 |
| 79 | 2946 | |  | | 0.156 | 0.03 | 0.173 | 0.137 | | 0.063 | 0.023 | | 0.027 | | 0.008 | | 0.045 | | | | 0.011 |
| 80 | 2951 | |  | | 0.09 | 0.021 | 0.324 | 0.266 | | 0.022 | 0.008 | | 0.014 | | 0.009 | | 0.021 | | | | 0.006 |
| 81 | 2968 | |  | | 0.043 | 0.009 | 0.173 | 0.147 | | 0.031 | 0.014 | | 0.023 | | 0.012 | | 0 | | | | 0 |
| 82 | 2990 | |  | | 0.08 | 0.012 | 0.266 | 0.196 | | 0.064 | 0.026 | | 0.02 | | 0.01 | | 0.06 | | | | 0.012 |
| 83 | 2998 | |  | | 0.389 | 0.136 | 0.599 | 0.305 | | 0.143 | 0.034 | | 0.064 | | 0.025 | | 0.24 | | | | 0.03 |
| 84 | 3018 | |  | | 0.219 | 0.054 | 0.39 | 0.21 | | 0.103 | 0.044 | | 0.035 | | 0.009 | | 0.375 | | | | 0.054 |
| 85 | 3038 | |  | | 0.275 | 0.036 | 1.037 | 0.408 | | 0.153 | 0.04 | | 0.055 | | 0.008 | | 0.539 | | | | 0.077 |
| 86 | 3048 | |  | | 0 | 0 | 0 | 0 | | 0 | 0 | | 0 | | 0 | | 0.078 | | | | 0.012 |
| 87 | 3058 | |  | | 0.12 | 0.025 | 0.053 | 0.02 | | 0.012 | 0.005 | | 0.017 | | 0.012 | | 0.078 | | | | 0.013 |
| 88 | 3059 | |  | | 0.069 | 0.028 | 0.069 | 0.046 | | 0.009 | 0.004 | | 0.013 | | 0.006 | | 0 | | | | 0 |
| 89 | 3073 | |  | | 0.063 | 0.009 | 0.198 | 0.152 | | 0.019 | 0.006 | | 0.015 | | 0.005 | | 0.069 | | | | 0.007 |
| 90 | 3095 | |  | | 0.128 | 0.019 | 0.1 | 0.031 | | 0.036 | 0.01 | | 0.024 | | 0.007 | | 0.073 | | | | 0.012 |
| 91 | 3102 | |  | | 0 | 0 | 0 | 0 | | 0 | 0 | | 0 | | 0 | | 0.027 | | | | 0.005 |
| 93 | 3119 | | 8.42 | | 1.078 | 0.989 | 0.085 | 0.04 | | 0.062 | 0.016 | | 0.028 | | 0.007 | | 0.043 | | | | 0.009 |
| 94 | 3139 | |  | | 1.177 | 1.131 | 0.074 | 0.049 | | 0.008 | 0.002 | | 0.015 | | 0.006 | | 0 | | | | 0 |
| 95 | 3169 | |  | | 0.303 | 0.07 | 0.148 | 0.055 | | 0.139 | 0.037 | | 0.085 | | 0.023 | | 0.08 | | | | 0.012 |
| 96 | 3178 | | 3.27 | | 0.175 | 0.036 | 0.024 | 0.006 | | 0.018 | 0.005 | | 0.019 | | 0.012 | | 0 | | | | 0 |
| 97 | 3188 | |  | | 0.271 | 0.072 | 0.051 | 0.011 | | 0.045 | 0.011 | | 0.055 | | 0.034 | | 0 | | | | 0 |
| 99 | 3214 | | 4.62 | | 0.214 | 0.084 | 0.072 | 0.024 | | 0.011 | 0.004 | | 0.007 | | 0.003 | | 0 | | | | 0 |
| 100 | 3226 | |  | | 0.487 | 0.338 | 0.014 | 0.006 | | 0.012 | 0.002 | | 0.026 | | 0.005 | | 0 | | | | 0 |
| 101 | 3237 | |  | | 0.055 | 0.008 | 0.033 | 0.004 | | 0.019 | 0.004 | | 0.013 | | 0.005 | | 0 | | | | 0 |
| 102 | 3253 | | 7.97 | | 0.185 | 0.03 | 0.059 | 0.01 | | 0.047 | 0.017 | | 0.038 | | 0.019 | | 0.071 | | | | 0.016 |
| 103 | 3270 | |  | | 0.156 | 0.028 | 0.094 | 0.034 | | 0.04 | 0.013 | | 0.023 | | 0.008 | | 0 | | | | 0 |
| 104 | 3273 | |  | | 0.026 | 0.003 | 0.015 | 0.003 | | 0.014 | 0.004 | | 0.012 | | 0.005 | | 0 | | | | 0 |
| 106 | 3304 | |  | | 0.525 | 0.451 | 0.031 | 0.007 | | 0.012 | 0.005 | | 0.014 | | 0.006 | | 0.093 | | | | 0.029 |
| 109 | 3371 | |  | | 0.217 | 0.046 | 0.098 | 0.026 | | 0.181 | 0.04 | | 0.219 | | 0.107 | | 0 | | | | 0 |
| 110 | 3381 | |  | | 0 | 0 | 0 | 0 | | 0 | 0 | | 0 | | 0 | | 0.921 | | | | 0.227 |
| 111 | 3389 | |  | | 0.092 | 0.016 | 0.072 | 0.024 | | 0.025 | 0.01 | | 0.018 | | 0.005 | | 0.149 | | | | 0.026 |
| 112 | 3395 | | 6.56 | | 0.062 | 0.01 | 0.047 | 0.006 | | 0.053 | 0.015 | | 0.033 | | 0.007 | | 0 | | | | 0 |
| 113 | 3420 | | 7.63 | | 0.024 | 0.004 | 0.038 | 0.016 | | 0.062 | 0.025 | | 0.018 | | 0.003 | | 0 | | | | 0 |
| 114 | 3427 | |  | | 0 | 0 | 0 | 0 | | 0 | 0 | | 0 | | 0 | | 0.399 | | | | 0.063 |
| 115 | 3434 | |  | | 0.059 | 0.01 | 0.057 | 0.029 | | 0.016 | 0.006 | | 0.012 | | 0.004 | | 0 | | | | 0 |
| 116 | 3448 | |  | | 0.073 | 0.019 | 0.106 | 0.057 | | 0.035 | 0.021 | | 0.265 | | 0.258 | | 0 | | | | 0 |
| 117 | 3461 | | 21.79 | | 0.102 | 0.022 | 0.052 | 0.005 | | 0.109 | 0.024 | | 0.068 | | 0.036 | | 0 | | | | 0 |
| 119 | 3535 | |  | | 0.066 | 0.01 | 0.107 | 0.05 | | 0.042 | 0.01 | | 0.03 | | 0.009 | | 0 | | | | 0 |
| 120 | 3556 | |  | | 0 | 0 | 0 | 0 | | 0 | 0 | | 0 | | 0 | | 1.107 | | | | 0.286 |
| 121 | 3561 | |  | | 0.477 | 0.111 | 0.2 | 0.037 | | 0.153 | 0.065 | | 0.197 | | 0.075 | | 1.475 | | | | 0.364 |
| 122 | 3575 | |  | | 0.187 | 0.027 | 0.136 | 0.038 | | 0.065 | 0.022 | | 0.05 | | 0.013 | | 0 | | | | 0 |
| 123 | 3598 | |  | | 0.425 | 0.067 | 0.245 | 0.03 | | 0.175 | 0.044 | | 0.091 | | 0.04 | | 0.4 | | | | 0.073 |
| 124 | 3623 | |  | | 2.62 | 0.702 | 1.584 | 0.291 | | 0.841 | 0.245 | | 0.413 | | 0.157 | | 1.183 | | | | 0.311 |
| 125 | 3634 | | 5.3 | | 0.282 | 0.091 | 0.787 | 0.124 | | 0.014 | 0.003 | | 0.058 | | 0.044 | | 0 | | | | 0 |
| 126 | 3641 | |  | | 0.183 | 0.053 | 0.231 | 0.042 | | 0.02 | 0.011 | | 0.075 | | 0.062 | | 0 | | | | 0 |
| 127 | 3648 | |  | | 0.147 | 0.04 | 0.16 | 0.034 | | 0.016 | 0.007 | | 0.067 | | 0.053 | | 0 | | | | 0 |
| 128 | 3659 | |  | | 0.117 | 0.032 | 0.062 | 0.012 | | 0.029 | 0.011 | | 0.035 | | 0.021 | | 0 | | | | 0 |
