# Supplementary material for: Cuticular Compounds Bring New Insight in the Post-Glacial Recolonization of a Pyrenean Area: Deutonura deficiens Deharveng, 1979 Complex, a Case Study
Source: PLoS One. 2010 Dec 21;5(12):e14405. doi: 10.1371/journal.pone.0014405 (PMC3014355; doi:10.1371/journal.pone.0014405)
Supplement: Table S2 — Discriminant analysis results between sexes in D. d. meridionalis (P<0.00001, F = 193,3723; Jackknife 100%). (0.03 MB DOC) [file pone.0014405.s002.doc]

Table S2. Discriminant analysis results between sexes in *D. d. meridionalis* (P<0.00001, F=193,3723; Jackknife 100%)

|  |  |  |  |  |  |  |
| --- | --- | --- | --- | --- | --- | --- |
| **Compounds #** | **Kováts' index** | **"F-to-remove"** | **Mean relative surface in Males** | **Standard Error** | **Mean relative surface in females** | **Standard Error** |
| 13 | 2035 | 42.34 | 0.192 | 0.035 | 0.246 | 0.068 |
| 15 | 2054 | 31.25 | 1.649 | 0.392 | 2.133 | 0.559 |
| 16 | 2075 | 97 | 0.12 | 0.014 | 0.199 | 0.048 |
| 42 | 2540 | 17.15 | 0.079 | 0.011 | 0.316 | 0.128 |
| 47 | 2606 | 12.69 | 0.176 | 0.025 | 0.384 | 0.09 |
| 94 | 3392 | 8.79 | 0.921 | 0.511 | 0.694 | 0.347 |
| 95 | 3403 |  | 0.673 | 0.445 | 0.533 | 0.285 |
| 97 | 3449 | 37.42 | 0.048 | 0.017 | 0.11 | 0.056 |
